# Surgery

**Published:** 1826-01

**Authors:** 


### Surgery.

1. *Parallele der Französischen und Deutscten Chirurgie, &c. &c. A Parallel of French and German Surgery, being the Result of a Journey made in the years* 1821 *and* 1822. By Dr. Frederick Augustus Ammon, Practising Physician in Dresden. Leipzig 1823. 8vo. fol. 483.
2. *Characteristiche der Französischen Medicin, &c. &c &c. A Characteristic of French Medicine with a Comparative Glance at the English.* By Dr. Johann Ludwig Casper, Practising Physician in Berlin, Professor of Medical Jurisprudence, &c. &c. Leipzig 1823. fol. 608, 8vo.

The first attempt at making a comparative statement of national surgery, was undertaken by M. Roux, of Paris, in 1815, in which year his Parallel of English and French Surgery made its appearance. It is a difficult thing for any man, but particularly for a Frenchman, to admit, that a thing can be better done in any other country than his own, yet it will be in the recollection of many of our readers that M. Roux conceded much to the superior skill of the London surgeons in many particulars : we say London only, because our large provincial hospitals were not visited by that author, he having contented himself to take the metropolitan as the representatives of all our other medical institutions ; his acquaintance there was just as correct as could be expected from a visit of fourteen days. Had the German been treated with as much candour, as the English surgeons were with politeness, it is probable that the books we are about to analyze would never have made their appearance, as Dr. Ammon, in the preface to his work, complains bitterly of the injustice done to his countrymen, and assigns, as one motive for his publishing the present parallel, the wish of rescuing German surgery and German surgeons from the unmerited censures of M. Roux ; censures which a better acquaintance with their language and their labours would have prevented.

Surgery, in Germany, may be esteemed a modern science, for although at an early period the names of a Purman, Heister, Theden, Siebold and Richter are to be found, names which must ever be mentioned with respect, it is only within the present century that the science has been zealously cultivated and raised to a grade which does not avoid a comparison with England or France. The cause of this is to be sought for partly in the pedantic prejudices which the physicians of former times entertained against surgery, and partly to the foreign influence which, for so many years, prevailed and depressed the native talents of the country. During the long and severe campaigns of Frederick, the importance of surgery was every where felt ; and on the return of the practitioners from the army to civil life, opportunities were afforded of reflecting upon the state of the science, and studying how it might be improved. It was at that time that a taste for human and comparative anatomy sprung up ; a new impetus was given to such pursuits, by the establishment of several periodical publications, devoted to these subjects, and the professional attention was kept alive to their importance and necessity, until the zeal with which they were prosecuted attracted the attention of the government, which, in its turn, did its duty by encouraging the cultivation of sciences so essential to the public safety.

In forming an opinion of the relative merits of French and German surgery, it is necessary to bear in mind the short time which it has been scientifically studied, the peculiar disadvantages under which the study has been prosecuted, and the great facilities which, for a longer period, have been afforded to it in France. Notwithstanding the appointment of Pitard to be body-surgeon to Louis IX, and the great honour conferred on Paré in the sixteenth century, in being permitted to shave his royal master's beard, surgery did not make much progress until the founding of the great hospitals of Paris, an epoch which must be esteemed the most important in the history of French medicine, as the "punctum saliens" of the profession in that country. From the time of Louis the Ninth up to the period of the revolution, this branch of natural science was peculiarly encouraged, and we are informed by Cuvier in his Eloges Historiques* that Peyronie of Montpellier, at that time the most flourishing school of medicine, was called to the residence of the court to give Louis XV. lectures on anatomy.

At the breaking out of the revolution, Paris, Montpellier, and Strasburg, were the three first medical schools of France, and the two latter far excelled in reputation the first. When the government of the kingdom became vested in the hands of Napoleon, his ambition prompted him to take every measure to make the capital of the empire, that which Cæsar made Rome, the capital of the world. Every corner of France was ransacked to find men who might give eclât to the Parisian University, and when found, it may be supposed that the arguments employed to bring them there, seconded as they were by temptation and by power, did not fail of effect. The fame of Strasburg and Montpellier sunk, as that of Paris rose, until the last soon outstripped the sister institutions. The political changes, which at that time occurred and which excited such destructive influences on most literary establishments, left those of Paris unhurt; they remained protected by the special care of the Emperor, and thus Paris became the seat of the science, as well as of the fashion of France. In addition to the protection thus afforded, in addition to the excellent opportunities of studying disease, which the large hospitals of the city afforded, the legal enactments at that time put in force, must be mentioned as having a considerable influence in raising the character of the profession in public esteem.

In France, however, notwithstanding the restrictive ordonnances to which we have just alluded, ignorant pretenders are allowed to insult the public by the announcement of their miraculous powers; there charlatanry flourishes in every possible way, and may correctly claim that country as its birth-place. In the streets of Paris, and in the provincial towns, one daily sees in the windows the inscriptions "*chirurgién herniaire, pedicure, et dentiste,*" over which is placed the head of Hippocrates or Galen, a molar tooth or a truss, and not unfrequently a list is superadded, containing an account of the many marvellous cures performed on Monsieur or Madame, with the liberal offer of communicating a complete knowledge of the art for a few Louis-d'ors in a few weeks. On the other hand, it must not be supposed that no traces of quacking remain to tarnish the rising reputation of German surgery; the ancient league, which appears to have existed in all countries between the surgeon and frisseur, has not been altogether broken, and you are yet permitted to see in many towns, particularly in the southern parts of the kingdom, the degrading advertisement "*Hier wohnt n. n. Schnitt und Wundarzt.*" The eye is frequently greeted by the glittering array of the seven brazen lather-basons suspended from the doorway of the pseudo-surgeon, and to this tabernacle of professional toleration, young and old, are invited to cast away their infirmities by the modest announcement, that the resident is a "*Meister seiner Kunst.*"

* Eloges Historiques, tom 2, p. 277.

From what has been said of the rapid rise of the Parisian schools, and the causes, we may consider them as the most perfect of France, and, therefore, in taking a glance at the existing state of medical institutions of that country, we may safely keep our attention principally fixed upon Paris. The school of Montpellier, it is true, still holds a respectable rank, but in all important particulars, the resemblance between it and Paris is so great, that it will not be necessary to refer to it. Wherever anatomy is cultivated zealously, surgery is seen to make progress, hence we find that degree of manual dexterity, to which some of the Parisian surgeons have arrived, may be traced principally to the plentiful supply of subjects always to be found at that school. The French students, however, are anatomists rather from the frequency of careless dissections, than from dissecting any part minutely; and one may spend a whole winter among them and not see a course of dissections made of any system, whether muscular, vascular, or nervous: the dissection of the vessels and nerves of the hand is commenced, or, perchance, it may be the fore-arm; but it seldom happens that the pupil begins at the shoulders, and traces the nerves and vessels to their extremities. But who ever attended a course of dissections at the Ecole de Médécine, or La Pitié, without feeling disgusted at the filthy and comfortless state of the rooms, where the hands are soon benumbed with the cold and the feet up to the ankles in water? But let a man cross the Rhine, and spend a winter at Göttingen or Berlin, at Breslau or Halle, and the change, in this respect, is very striking. The state of the buildings in which so many hours must be spent in the most dreary part of the year, is more calculated for comfort and convenience. The rooms are, in general, spacious and well ventilated, dry and clean; and at Göttingen in particular, the anatomical student has the greatest regards paid to his comfort; the room is not so large as that at Berlin, but is sufficiently large for the number of the students, and, in severe weather, it is well warmed with stoves. The floor, the walls, and the tables, all give proof of the great attention to *Reinlichkeit;* the subjects are well injected, and the parts to be dissected are first put into spirits of wine. The whole room is so neatly arranged, that it is not uncommon to see the student with his long pipe and coffee by his side, whilst, with the hook and scalpel, he is tracing the minute anastomosis of an artery, or the delicate distribution of a nerve. No doubt the pupils are principally indebted, for this state of the dissecting room, to the personal attention of Langenbeck, who is daily to be found among them taking the greatest interest in the science, and as zealous in the pursuit of it as if just entering on his professional career. The dexterity of Langenbeck, as a surgeon, is known throughout Europe, and great as his reputation deservedly is, he is so warmly attached to anatomy and so sensible of its importance, that he said, in a conversation which turned upon this subject— "If I am asked which of the two studies I would relinquish, surgery or anatomy, I should answer that I would rather never more take a knife in my hand, than give up the pursuit of a science so interesting and important as anatomy." It might be supposed that subjects at Göttingen would be scarce, and so they would be if the University had to trust to the town for the supply, but many are secured from the adjoining parishes, so that the supply is sufficient for the wants of the pupils. At Berlin, the Charité furnishes even more than is necessary, and nothing is wanting there to make the anatomical department equal to that of Göttingen, but a greater degree of attention from the teachers to the taught. Knobe and Rudolphi, the professors of anatomy, visit the rooms frequently at the commencement of the session, but their attendance soon becomes irregular, and, towards the close of the semestre, their pupils who are industrious enough to remain there so long, are seldom favored with a sight of their illustrious preceptors. No demonstrator is appointed, so that if the friendly habits of the pupils did not keep up the Lancasterian mode of instruction, they would be left to explore their way as well as the "*Handbuch der Anatomie*" would enable them. In some of the southern German universities, a deficiency is occasionally felt, but it can never be said to amount to an absolute scarcity. On the whole, we may observe that the German pupils dissect less than the French, but what they do they do better.

The only institutions in Paris at which any thing like clinical instruction is afforded, are the Hotel Dieu, the Charité, and the Clinique de Perfectionnement. At the first, presides Dupuytren; at the second, Mayer and Roux; and, at the third, Dubois, undoubtedly the first practitioner in Paris. This, observes Dr. Ammon, is the only institution worthy the name of a clinique. The pupil has there an opportunity of observing the various characters of external, as well as of internal diseases. The lively interest which this much respected man takes in the instruction of the pupils, the pleasure shewn when pointing out the different forms of disease, shew how anxious he is that the pupils should be well instructed. See, said Dubois to his pupils, "ces êtres souffrans, (les malades) sont vos livres, vos maitres, les veritables instruments de votre instruction," spoken with so much energy, that none who heard it could remain uninterested in the science of which he was speaking. With respect to the clinique at the Charité or the Hôtel Dieu, the only persons who are there likely to profit are the *élèves internes*, on whom the duty devolves of securing fractures, reducing dislocations, stopping hæmorrhages, &c. and, by their frequent opportunities of visiting the sick, they only observe the effects of remedies with accuracy, whilst the *οἱ πολλοὶ* of the *élèves externes* go through the wards like so many pieces of machinery, without gleaning a single idea of any practical utility. As to Dupuytren, he seldom speaks, except to abuse a dresser (elève interne) or a nurse. On the history, present state, or probable termination of the patient's case, he seldom utters a word; and it is with regret that we are obliged to add, that the conduct of a man, whose name ranks so high in the profession, should be, towards his pupils, more like the demeanour of a general to his soldiers, than that of a teacher to whom they look up with reverence and respect. What can be gained by running round the wards of a hospital with a snarling surgeon, who so far occasionally loses sight of the conduct which one gentleman should observe to another, as to express his disapprobation by a kick on the shins, or a "*donnez vous place.*"? Attendance on hospital practice, as it is called, in this country, is one of the principal means which a young man has of studying his profession, and what can he be expected to know if the persons on whom he relies for information, should seal up their mouths in sullen obstinacy, or deliver their opinions in such dogmatic confidence as to deter a pupil from asking a second question? On the contrary, in Germany the case is quite different, and Dr. Ammon may, with great propriety, congratulate himself on the manner in which clinical education is conducted in his native country. At every university is a hospital, or wards of a hospital, set apart for the clinicum, into which such patients are brought as present something worthy of notice, and in whom the characteristics of certain diseases are well marked. The pupils are not allowed to wander about as if they were strangers paying an occasional visit, but are induced to take a lively interest in the progress of each case, by being questioned, on one visit, what treatment was adopted at the last, and the reasons for which such treatment was adopted. At the bedsides, the pupils are publicly asked to give their opinion of the patient's case, and to state the reasons on which that opinion is founded; one may be desired to enumerate the leading symptoms, another to give the varieties which the disease presents, and a third to point out the diagnosis between it and the disease to which it bears the nearest resemblance. The answers given are commented on by the conductor of the clinicum, and if he differs in opinion from the pupils, he states his *reasons* for doing so, these generally amount to *seven*, sometimes to more; but if a German cannot enumerate seven reasons for doing or believing, he is set down as a superficial thinker. The remarks made upon the case by the surgeon are useful, although somewhat more diffuse than is necessary, so that a clinical ward is made of more utility than the range of a large hospital, and every case is made a lecture.

It must be expected that the clinicums in the second class of university-towns are small, but they are, in general, well managed and productive of great practical utility. It is a system which cannot but be praised, and it is only to be regretted that this mode of pursuing a medical and a surgical education is not more closely imitated in this country. If we may be allowed to suggest a plan for the introduction of such a system into our metropolitan hospitals, we should recommend the following:—To set apart wards, as clinical wards, would not answer in the large hospitals, because the concourse of pupils is so great that it would be impossible for each pupil to hear what might be said, much less to get a sight of the patient. The crowding round the beds of the sick in a dense mass can be productive of no good, but is frequently productive of much evil; such a practice is very inconvenient to the individual whose opinions the students are anxious to hear, alarming to the patient, and an annoyance to the whole ward. As a substitute for this practice we recommend the following, which is a brief description of Graefe's clinicum, at Berlin. Near the wards is a circular amphitheatre, having an area sufficiently large to contain a bed, and to admit of five or six persons standing round it at a little distance, so as not to intercept the sight of the patient from the pupils, who are ranged in circles gradually ascending to the sixth. The patient, if not able to walk in, is brought on his bed, carried by two men with poles, like a sedan-chair, and placed in the centre of the area. If this should be the first time that the patient has been shown, Gräefe asks such questions as are necessary to let the pupils hear the account which the patient can give of the present state of his complaint, a short account of its origin and progress, and he is then removed to the ward slowly and carefully, as he was brought into the theatre. If the case should be such as will probably require an operation, it is confided to the care of a second semestre pupil, who has to write a history of the case before the next clinical meeting, which is in two days, either in Latin or in his own mother tongue, and describe the origin, progress, and present state of the disease, the diagnosis, prognosis and treatment, and state why an operation is necessary, or what chances remain of its being avoided by the adoption of other measures. This is read before all the pupils, who preserve the greatest order and evince the greatest attention. Gräefe then makes such remarks on the case as he deems necessary, points out any error which may have occurred in the description, and corrects any fallacy which may have crept into the conclusion. The patient is then brought in, and thus an opportunity is afforded to every one present of observing whether the case has been accurately delineated. A case of this sort is introduced at every clinicum; and, in addition to this, two or three other cases are brought in on which the pupils, who are with Gräefe in the area, and who take it in turns to be there, are asked their opinions and how they would treat them. An interest is thus excited, and each pupil is obliged to make himself thoroughly acquainted with a case, in order to support a reputation with his fellows. But we come now to the peculiarity of this institution, the permission given to pupils who have given good proofs of their competency to perform operations under the immediate superintendance of Gräefe. The various needle operations on the eye, fistula lachrymalis, hydroceles, fistulæ in ano, amputations of the fingers, toes, &c. are all performed by the pupils, an opportunity afforded at no other institution except to the military surgeons at the Charité. This was formerly allowed at Vienna, when the ophthalmic department of the great hospital was under the management of Bëer, and is still permitted at Florence! indeed there the surgeon never performs any operations, all being done by the pupils. RUST's clinicum at the Charité, although differently managed, is a very excellent institution; the practical tact which he displays and the good humour with which his observations are blended, cause him to be esteemed as a teacher and respected as a man of science. There is an ingenuousness of appearance about him which never fails, at the first sight, to please, and the honest independence of character, which a more intimate acquaintance with him affords, confirms the favourable impressions first produced in the mind of the stranger. LANGENBECK's clinicum at Göttingen, and the industrious WALTHER's at Bonn, although on a smaller scale, are equally good in their kind, and are the nurseries of German surgery. Professor WATTMAN's, at Vienna, must not be omitted, which is even larger than Rust's or Gräefe's and equally well conducted.

Professor CASPER has given in his book* a good sketch of the characteristics of French medicine. He has spoken with becoming respect of the French physicians, and whilst he has deservedly given his meed of praise to such men as Recamier, Lerminier, Fouquier, Meckel and Laennec, he has not spared their foibles nor refrained from expressing his opinions freely on the *citelkeit* of the French character. The rapid transition from one system to another, the premature adoption of new hypotheses, the prejudices for particular medicines, all tend to prevent the study of medicine from arriving at any great profundity. The doctrine which prevails to day is effaced by the novelty of to-morrow. The whole of the profession of France has been successively set by the ears with the dogmas of Brown, Rassori, and Broussais, but at the present time, the general practice of medicine is very inert, and we may strike a balance between the contending claims of the advocates of stimulation and contra-stimulation, and say that a tisan and a leech are as much as it amounts to. The doctrine that "La connaissance de la gastrite et de la gastro-enterite est la clef de la pathologie," is fast giving way, and will, in all probability, in a few years be laid aside for another. Medicine has undergone more changes in Germany than in any other country, not even excepting France; this is partly owing to the philosophical turn of the German character, which leads readily astray into the depths of speculation, and partly to the multiplicity of books, which are greedily translated from foreign countries, full of crude, indigested materials. The words *organismus*, *constitutio atmospherica*, *constitutio humana*, and *magnetismus* are on every German's tongue, and serve to render unintelligible the most self-evident truth, and to veil, in erudite obscurity, the profundity of German reasoning. The doctrine of animal magnetism, which, two or three years since, was becoming very popular, not only in Berlin, but throughout Germany, has now sunk into the quiet of oblivion; and Dr. WOLFART is content to give a *publice* on *semioticca nosologicum et therapeuticum* sententiis Hippocraticis illustratum duce compendio suo (Grundzuge der Semidtik etc. Berol, 1817)! which nobody attends, and a *privatim*

* Chap. iii. fol. 39, et seq.

on *nosologiam et therapiam specialem*, at which he has about a dozen pupils. The story which has been told of the Doctor by a late tourist, is much exaggerated, yet there is little doubt that advantage was taken of the credulity of a female to make a breach in her virtue, a crime, for which all the maids of the animal magnetismus can never atone. In their practice, the Germans, although not so bold with the use of the lancet as the English, are much more courageous than the French; they still retain great prejudices against cold air and cold drinks, and in their prescriptions one yet sees too much of the "copia rerum" of the antient compounders.

We cannot stay to trace the influence which the prevailing medical theories have had on the surgical practice; a subject which both Drs Casper and Ammon have investigated at considerable length, and, with the characteristic taste of their countrymen for abstruse subjects, have occupied many pages in debating pro and con, to settle a point which after all can only be speculative. We shall rather apply ourselves to the practical part of the work, and having brought down the history of surgery in general, and of the institutions for surgical education in this and other countries, and taken a glance at the present state of medicine; we shall have reached that part of the first book in which the author speaks of the application of fire in the treatment of surgical diseases.

Moxa and the actual cautery have been used frequently, both in France and Germany. Rust has tried the moxa more frequently than any other surgeon in Germany, and he says that he has not found it answer the expectations which had been raised of its peculiar efficacy.* In France our readers are aware that it has long been a common remedy. Aulagnier, in 1805, published his researches on the employment of fire in diseases reputed incurable.† Since which time a host of French writers have succeeded, but as we have lately had occasion to notice this subject, it will not be necessary to enumerate them. In Germany, moxa has not been so generally used as in France, and this circumstance arises not from any dread which the surgeons have of the remedy, or that they consider it too severe in application, but from a preference which they have for another yet more terrific remedy, at least it appears so to a man who has received his surgical education in England. Dr. Ammon is a patron of the actual *ferrum candens*, and thinks that its efficacy in *coxitis*, is superior to that of the moxa. He says that he has seen the ferrum candens, used in several cases of white swelling of the knee in the clinicum of one of the first surgeons of Germany, and that the removal of that afflicting disease has been more rapid than by the application of moxa, in the same cases in the Charité of Paris, at which Roux, who is a great friend to moxa, is the second surgeon. We have also had occasion to see the application of the "ferrum candens" in the Clinicum, to which Dr. Ammon alludes in cases of chronic scrofulous inflammation of the knee-joint, and also of the hip, and to say that no benefit was derived from the treatment would be saying what is wrong, but we doubt much whether more good was done than might have been accomplished by more lenient measures. There is something very repugnant to the feelings, in seeing a red hot iron burning its way slowly into a man's hip, frizzing and roasting the gluteus maximus, and causing a cloud of dense stinking smoke to fill the apartment. The iron used there, is of a circular figure, about an

---

* Vide Rust's Arthrokakologie od. iib. d. perrenkungen durch inuere Bedingungen und iib. and Heilkroft, wirkungs und anuendungsart des Gluheisens berg diesen Krankheiten. Wien, 1817.

† Recherches sur l'Emploi du Feu dans les Maladies reputées Incurable. Paris, 1805.

inch in diameter, and an inch and a half long, appended to the end of a piece of stout iron wire which serves for the handle: it is a farriery-like practice, and we hope will never be revived in this country.

Another point of treatment in which the German surgeons differ from the French, is in the application of arsenic to sores. The French use arsenic very plentifully, both internally and externally, to scrophulous, syphylitic, phagedenic, and cancerous ulcers. The form in which it is most frequently employed is that of the *pâte arsenicale,* which is made with the powder of Rousselot,* mixed with a little water, and then spread over the diseased surfaces. Dupuytren generally uses a powder composed of 0,98 calomel, and 0,12 arsenious acid. The French physicians are just as much attached to the administration of arsenic, as the surgeons are to its external employment. CASPER, in his Chap v. has given a good account of the effects produced by this medicine in several cases of diseases of the skin, epilepsies and agues, which came under his observation during his residence in Paris, and of which we regret that our limits will not allow us to give a more extended notice. The German surgeons use the arsenical ointment, a form particularly recommended by Rush, in cases of cancerous sores of the lips, and intractable ulcers in various parts of the body, but this to a very limited degree when compared with French.

The treatment of fistulæ has been found in all countries to puzzle the ingenuity of the surgeon, and we cannot, therefore, wonder if the author joins in the common lamentation over the uncertainty of the art in removing them. Compressions, stimulating injections, the seton and the knife, have been in their turn employed, and each may be said to hold a place in the practice of modern surgery. Langenbeck was the first surgeon who drew the attention of his countrymen to the mode of curing fistulæ by passing a ligature through the fistulous canal; but it must not be supposed that this is any novelty, for we find that our old friend Celsus distinctly recommends the practice.† In a case which recently occurred of fistulous suppuration of the hip-joint followed by ulcerations of the ligaments, and dislocation of the bone upon the ilium, Dr. Ammon relates that he saw the fistulous ulcers, which had formed among the muscles of the hip, successfully treated, by the introduction of a seton, with Potts' needle and canula. In a few days, the discharge of matter, which had been very considerable for more than a year, ceased, and the patient, whose death was daily threatened, restored to health. In France, RIBES and LARREY, and most other surgeons, operate with the knife; and, although REISINGER has lately made an attempt to revive the use of the seton in the treatment of fistula, the greatest number of surgeons use the knife. Whatever mode may be employed, the object of all operations for fistula is, to heal them, by a process of granulation, from the bottom; and it is impossible to say that one shall always be used to the exclusion of the rest. The best accounts we have seen of the rules for the application of the various modes of treatment of vesico-vaginal, vesico-rectal, and urethro-rectal fistulæ, are to be found in Chelius‡ and Schreger,§ and in Richerand's Nosographie et Therapeutique Chirurgicales, a work well known to the profession of this country.

On the subject of fractures, it would, indeed, be a difficult thing to lay down any treatment as being generally adopted: there is, perhaps, in Ger-

* Rousselot powders consist of 0,70 d'oxyde sulfuré rouge de mercure, 0,22 sang. draconis, 0,8 oxyde blanc d'arsenic.

† Celsus de Ani Fistuli, lib. vii. 4.

‡ Chelius' Handbuch der Chirurgie, &c. fol. 509, *et seq.*

§ Schreger's Grundriss der Chirurgischar Operationen.

many, on no point of surgical practice, so much difference of opinion, as on this, and to attempt to describe the numerous mechanical contrivances used in the management of fractures, would be to impose a duty upon ourselves which we could never perform, as almost every surgeon has his peculiar fracture-box, embracing something which he deems an improvement on that used by his predecessor or colleague. The best proof, however, of the utility of any machine in the management of a fracture, is the degree of restoration of the injured part with which it will permit the patient to escape from a tedious confinement. If a limb can be secured with a couple or three splints, and secured firmly, we would use them in preference to all screwing, bandaging, lacing and compressing, which the ingenuity of man can produce; the more simple the mechanical contrivance is, the better, and this holds good not only in the various productions of art, but, also, where we wish to assist the reproductions of Nature. In the Hotel Dieu of Paris, is a better opportunity afforded of seeing every form and complication of fracture, than at any other institution in the world; the situation of the Hospital, surrounded as it is by narrow streets filled with a busy and active population, gives it this advantage. In it, a ward is set apart for the reception of fractures only, which has been, for many years, the repertorium of these accidents. DUPUYTREN, during the many years which he has had the superintendance of this Hospital, has put into practice almost every method of treating fractures that has been suggested by persons of all countries, and is far, even at present, from adhering to any determined mode of treatment, but adopts different means according to the requirements of the different cases. Fractures of the fore-arm are treated by him, and, indeed, by most other French surgeons, by passing a circular bandage around the arm, beneath which, between the radius and ulna, is placed a graduated compress, so as to keep the ends of the bone in perfect apposition. The fractures of the humerus, as well as the fracture last-mentioned, are managed alike in all countries in all important respects, and it will not be necessary to go into detail of the history of splints and bandages. LE DRAN, MUSCATI, and DAVID, have each had their turn, and each retains some advocates. The manner of using their apparatus, is well described in that useful publication, the Memoires de l'Academie de Chirurgie, tom. iv.; whilst, in later times, BOYER, CHAPEL and REYNAUD, have each cut a conspicuous figure. BOETTCHER* and BRUNINGHAUSEN† have been the principal persons who, in their time, took the lead in Germany: it is only necessary, however, to state, the modern German surgeons have far outstripped their predecessors in the treatment of fractures. The principles generally observed, and the practices which are more extensively adopted than any other, are to be traced up to Dessault, and he is, therefore, entitled to whatever of merit they may possess.

In France, as in Germany, many surgeons doubt the possibility of fractures, transverse fractures of the patella, uniting by a deposition of callus. LANGENBECK is, however, the advocate of the contrary doctrine, and contends, that fractures of the patella can, and do frequently unite by bone and not by ligament, when properly treated;‡ whilst DUPUYTREN, in France, stoutly maintains the same, and confidently refers to a preparation now in his own possession as setting the possibility of union beyond all

---

* Boettcher, J. R. Auswahe des Chirurgischar, verbundes far aigisrinde Waudarzte 1795.

† Uebn der bruch des Schlussenbein.

‡ Vide Langenbeck's Neue Bibliothek fur Chirurgie und Ophthalmologie, b. iii. chap. i. fol. 49.

dispute. These two eminent surgeons, with some others in this country, assert that it is only necessary to leave Nature undisturbed, by removing any thing which may lead to a separation of the fractured ends of the bone, in order for Nature to accomplish a bony union. The time which Dupuytren considers necessary for a union of the fracture of the olecranon is fifty days, or, at least, he never suffers the bandages to be removed within that time. The time which the patella and the fractured neck of the femur must remain secured is much longer, the time varying from 90 to 110 and 120 days, and sometimes, indeed, to a longer time. Of the modes employed by Dupuytren and Langenbeck, it would occupy too much space here to give a minute account; we wish only to state the fact, that they deem the bony union of the transverse fracture of the patella possible, where proper mechanical powers are resorted to, in order to retain the fractured ends of the bone in correct apposition. Respecting the question of the possibility of union of the fracture of the neck of the thigh-bone within the capsular ligament, Dr. Ammon has the following paragraph.* "Many English surgeons doubt with Casper, the possibility of a perfect union, by bone, of a fracture of the neck of the thigh-bone internal to the capsular ligament. (Fractura interna colli ossis femoris.) The French surgeons, however, with the German, are convinced, from pathological preparations, of the possibility of the union, and sincerely regret, that the propagation of a contrary opinion has caused the examples of a complete union comparatively rare, by inducing surgeons to give up that careful treatment by which alone the restoration is possible." In the pathological anatomical museums of Paris and Strasburg, are said to be six clear and distinct preparations of union of fracture of the neck of the femur within the ligament. In the pathological collection at Brunswick, it is said that an unequivocal proof of union of the neck is to be found, and that the case has been satisfactorily proved to be a united fracture, by having made a longitudinal inspection of the part; we cannot speak of them, however, from personal inspection, and, until we have an opportunity of doing so, we shall defer saying any thing further on the subject.

To form an opinion on the general state of operative surgery in France and Germany is an attempt of no easy execution. If we look at the schools which exist in the two countries for instruction in this branch of medical science, we find that they are nearly alike; and if we look at the manner of performing operations by the surgeons of both countries, and the degree of dexterity with which the most eminent in each perform their operations, we find that the shades of difference are slight; and it is only by entering into the merits of the plans of operators that any general inference can be drawn as to their relative superiority. There is, however, one striking distinction between the operative surgery of the two countries which cannot escape the attention of any individual who has had the slightest opportunity of observing, namely, the simplicity of the French, and the complication of instruments in the German operations. Whilst a French surgeon will only use a knife and a saw for the removal of a limb, and trust to an assistant to secure the principal artery, the German will employ two tourniquets, three knives, one for the incision of the integuments, another for the muscles, and a third for the periosteum; in addition to which, he pushes a retractor between the two bones of the leg or arm, and wastes time in fitting and adjusting it. Whilst a French surgeon will perform the operation of lithotomy with a knife, or only with the knife and the bistourie caché, the German uses half a score of instruments; first a knife for the division of the integuments, a second to divide the prostate gland, a third instrument to dilate the bladder, or rather

* Vide Dictter Abschnitt, fol. 154.

the incision made into the bladder, a grooved gorget for the introduction of the forceps, and so on, multiplied almost to infinity. A French surgeon contents himself with making a knife of one shape do for most amputations, the Germans have invented particular knives for the removal of the different parts, and not only for each part, but for the different dimensions of the part, so that the knife employed for the removal of the leg of a stout man, would not be used to remove the extremity of a spare one. A German's "*armentarium chirurgicum*" would be as astounding and unintelligible to a Frenchman as a German's logic. The ingenuity of the latter has not confined itself to the invention of useless instruments, but has displayed itself also in a ridiculous degree in the production of complicated modes of operating. Thus, a celebrated surgeon, who has made considerable stir in the staphyloraphy operation, strongly recommends a kind of chissel or guillotine, for the chopping off the edges of the cleft palate, and silver screws for the fastening of ligatures, instead of simple knots, with which an unsophisticated English surgeon would have been perfectly content. Instances might be multiplied without number, in which the most simple operations are disfigured by the complication of the instruments employed, and this practice cannot but be considered as a reproach to German operative surgery. From this censure, a few of the German surgeons must certainly be excepted, and particularly Langenbeck, who has raised his voice against this prevailing error. Alluding to the manner in which his countrymen perform the operation of venesection with a spring fleam instead of a lancet, this celebrated surgeon observes—"It is an indisputable truth, that the more simple the instrument, the better it is; it is not the mechanism of the instrument, but the hand which directs it, that is to perform the operation."* The application of bandages is made the subject of a distinct course of lectures, and it may be supposed that a professor of bandages will refine away a few pieces of tape and canvas to a never ending combination of figures, and assign to each becoming names upon Greek and Roman authorities; between each, a distinction without a difference is made to exist. Sutures, and other adjuvants to surgical practice, are equally profound, by puzzling, and it would be a lesson to any London student which would occupy him a week, to learn the names of the various bits of bobbin and steel which are tucked through the periphery of the human frame.

We shall now proceed to that part of operative surgery with which it becomes every surgeon to be familiarly acquainted. No country of Europe has been engaged in so many wars as the French, and, consequently, their surgeons have, before those of any other nation, had the best opportunity of studying the best mode of performing amputation in the fullest extent. Yet the difference of opinion on this subject is still very great, even among the French; not that there is any difference of opinion as to the indications and contra-indications for the performance of amputation, but on the manner only in which the operation shall be performed. The circular incision is the mode of operating generally adopted in Paris. Alanson, in proposing the conical incision, opened a new field for experiment, and the practice was violently opposed by Mynors, who thought he could succeed best by first saving the integuments, and afterwards cutting through the muscles higher up, a practice which, after a time, obtained considerable reputation.

Dessault's operation is only a modification of Mynor's, since the flap ope-

* "Es ist eine anomstössliche wachcit—je imfacher ein instrument, desto vesser. Nicht der mechanismus et des instrumentes, soudern die das instrument fuhrende Hand sole operiren."—*Langenbeck's Nosologie et Therapié der Chirurgischen Kraufhicten, Erster b. fol.* 690.

ration of the thigh was sometimes performed by him. The use of the long straight knife continues to the present day, and has many advocates. Dupuytren operates with the straight knife, and divides the integuments, in almost every operation, by the circular incision, which he makes at one sweep, and not at two as recommended by Langenbeck and Lisfranc. This is properly Louis' operation, and the only difference between it and that practised by Dupuytren at present, is this ; Dupuytren uses a straight knife, whereas Louis used one of a falciform figure. The amputations performed by this surgeon, although executed in a short time and with considerable neatness, do not reflect great credit on him, as in the greater number of cases, observed by Dr. A. the stumps afterwards became conical, the bones projected, and the patients frequently sunk from the irritation thus occasioned. In Germany, the circular incision is practised nearly as frequently as the *(lappen schnitt)* or flap operation, the latter being used as with us in such cases in which the integuments on the front of the leg are diseased too high up to admit of the circular incision. The great point on which the German and French surgeons agree, and in which they entirely differ from us, is in their healing their stumps *per secundam*, instead of *per primam intentionem*. Larrey and Dupuytren adhere to the practice of healing the stumps by the second intention, and whilst such men adopt that method, it will, of course, be followed by those who receive their surgical education from them. RUST, GRAEFE, WALTHER and others, follow the same rule, and thus the practice is perpetuated in both countries. In Germany, however, the circumstances which influence the mode of healing the stump are these ; where suppuration existed before the amputation was performed, as in cases of diseased joints, diseased bones, &c. it is considered necessary to heal by the second intention, whilst, on the contrary, if such a mechanical injury should be done to the limb as to require an amputation, then it is considered lawful to attempt the cure by the first. It may be supposed that the stuffing the hollow of a stump with charpie, and allowing a large cup to fill up by granulation, must be a very slow and painful process; of the latter, we are quite satisfied, but we have seen many stumps, in young men and children under twelve years of age, perfectly healed in fifteen days, and frequently within the three weeks.

The removal of the four fingers from their connexion with the metacarpal bones has been imitated by MAINGAULT,* who has frequently removed all the toes at their articulations with the metatarsal bones. The finger is first placed upon the upper part of the articulation of the great toe with the inner metacarpal bone, and, having ascertained precisely its situation, a long narrow knife, about the size of a common catlin, is taken, with which the joint is cut open towards the next toe, by describing somewhat of a semicircular incision from within ; outwards, every joint is laid open in succession. When the ligaments are all cut through, the edge of the knife is carried close round the heads of the detached bones and a flap cut out beneath, by carrying the knife carefully forward as far as the separation of the toes, when the knife is turned downwards, and the flap is thus perfected.† The dexterity of the French in removing fingers and toes is generally admitted, but the practice of amputating other parts at the joints is at present questionable ; the operation which we have just described appears well fitted for such cases in which the toes are severely frost-bitten, and in which the injury has not extended beyond the second phalanges.

Among the other operations which Dupuytren so skilfully performs, those

* Vide Maingault's Medicine Operatoire. Traité des diverses Amputations qui se Pratiquent sur le Corps Humain. A Paris, 1822.

† Maingault—planche v. fig. 20.

deserve to be named which have for their object the removal of the nails when they grow beneath the flesh (ongles entré dans les chairs.) Trivial as this operation may sound to the ear, it is one excessively painful to the patient and puzzling to the surgeon. The evil generally arises from the practice of wearing tight shoes and boots, and is of frequent occurrence. The sprouting of a fungus from the side of the nail was common in the days of Fabricius, ab aquapendente, who recommended an operation for its removal, which was improved upon by Dessault, and has been used also by Richerand, Guillemot, and others, but which seldom produces a radical cure. The method employed by Dupuytren is simply this; a straight scissars is suddenly pushed beneath the nail, near the edge of the fungus, with which the nail is divided longitudinally from the top to the bottom; the part of the nail to be removed is then laid hold of with a forceps and turned suddenly back, by which manœuvre, it is easily separated from its connexion with the parts beneath, and, with a scissars, removed at its base. The operation thus performed is not so painful as might be expected, and is, when dexterously performed, the best treatment to which the surgeon can have recourse. The fungous excrescence around the sore is touched with the actual cautery, but we think a little nitrate of silver would do as well, and thus the cure is performed. It is but justice to add, that the celerity with which Dupuytren performs his operations is equal to that of any, and superior to most of his cotemporaries.

The state of ophthalmic surgery in France and Germany exhibits a striking contrast, and if the latter country could claim no superiority in any other respect, it might certainly do so in this. That ophthalmology has not been extensively studied in France is a fact sufficiently notorious; it is not only observed by strangers, but it is confessed by the French surgeons themselves, that this branch of surgical science has been neglected, and if, in addition to this confession of inferiority, we could see that the French surgeons were, at the present time, taking any effective measures for the improvement of their ophthalmic surgery, we should refrain from acceding entirely to the justness of the censures of the author on their practice. We shall notice briefly the origin and progress of ophthalmic surgery in Germany, and then mention the measures at present in force for its advancement. The founding of the Vienna School, by the Empress Maria Theresa, in consequence of the zeal and ability displayed by Barth in the treatment of the numerous blind persons which he found in that city, must be considered as the first step towards raising ophthalmic surgery to a scientific point; it must not only be considered the mainspring of its cultivation in Germany, but also throughout Europe. What Barth and Richter begun and advanced so much, has, by the industry and talent of such men as Beer, J. Schmidt, Himly, Von Walther, Langenbeck, and Graefe, been raised to a great degree of perfection; by their efforts, a mass of information has been extensively diffused on a most interesting class of diseases, which has been attended with the greatest benefit to mankind. The number and the worth of the communications made to many of the existing journals, some of which are expressly devoted to this subject, show that the interest taken in the promotion of the science has not diminished, and the number of institutions set apart for the reception of patients having diseases of the eyes, are sufficient to afford ample opportunities for observation to those who must hereafter supply the places of the present practitioners. The men who are most skilled in the diagnosis of the various diseases to which the eye is subject, and who have most tact in the execution of the necessary operations, are those who consider the eye as correctly coming within the pale of surgery: these are the men who have most distinguished themselves and advanced ophthalmology, and who have, at the same time, shewed themselves the best surgeons.

In France, however, we do not find ophthalmic surgery in so flourishing a state, the causes of which are plain, and may easily be enumerated. *First,* We find no institutions established for the reception of patients having diseases of the eye, but we find them scattered up and down in the wards of the large hospitals, exposed to all the prejudicial influences of light, heat, and impure state of the air, where it is impossible to attend to their wants and to the state of the organ, so minutely as its delicacy and importance require. *Secondly,* We find that the pathology of the organ does not receive so much attention, is not considered of sufficient moment to entitle it to the special notice of the teacher, at least, we find that no course of lectures in the famed *Ecole de Medecine* is appropriated to this important subject. It forms a part of the course of external pathology only; and every well informed man must confess that it is impossible to do justice to ophthalmic diseases in a course of lectures embracing any other. *Thirdly,* The practice of this branch of surgery is confided to the hands of men, who are either not surgeons at all, or, if they have the name, possess that only, and are ignorant of all principles of the science, and barefaced empirics in their treatment and practices. It is to be regretted, that even well informed men, men of education, and who hold a respectable rank in society, should degrade themselves so much as to allow their names to be stuck up in the Coffee-houses and in the corners of the streets. It was very common to see the announcement of some wonderful feat performed by M. B. or M D. who, with *pommades antiophthalmiques,* promised to cure every thing to which it was applied. Not long since a *M. G. de la Chanterie celèbre medecin oculiste* regularly favoured every stranger on his arrival with the list of the astonishing cures which he performed, and this contribution to science was adorned with a drawing of the eye, on which the operation of extraction was performed according to Wenzel, over which was written in allusion to the knife, *rien sans lui,* and the whole concluded with the modest entreaty "*On est invité a donner la plus grande publicité au present avis!*" Another oculist, as he styled himself, who is at the present time esteemed a very clever man, and retains a great many patients, was in the habit of advertising to cure cataracts without any operation, which, according to his own account, he frequently accomplished by applying a hot iron to the back of the head, the "cauterization occipitale." In the provincial towns of France, the same practices prevail, as any one who has travelled through Nantz, Dijon, Chalons, &c. must have seen. The existence of such practices comes in corroboration of the low state of French ophthalmic surgery. Of late years, a few individuals have shown a little zeal for this department of surgery, and it is due to DEMORUS, GUILLIÉ and others to say, that they do not come under the censure which we have, in a former paragraph, expressed at the conduct of some of their cotemporaries. It must be also admitted, that however laudable their zeal may be, it has not yet produced much for the advancement of ophthalmology. As far as regards the French literature of ophthalmic diseases, there is certainly no want of new books and compendia, in which the diseases of the eye are treated apart from that of other surgical subjects, and as the best of their new books, we may mention DELARUE's and DEMOURS'; but we find, on comparing them with the works of their older authors, as GUILLEMEAU, MAITRE JEAN, ST. YVES and GUERRIN, that their ophthalmic surgery is not what it was in the last century. Of DEMOURS' book, which may be deemed the standard of this subject in France, Traité des Maladies des Yeux avec des planches colorées d'apres nature, 1818, in three volumes octavo, we may safely say that it contains no accurate description of diseases nor any information important in practice. The minute descriptions, necessary to distinguish accurately the various shades of difference in ophthalmic diseases, is lost sight of, and insignificant verbiage substituted for practical information. Long cases which no one will read, and a series of questions and answers which lead to no results, form the greater part of the work. The plates are just as bad as the other part of the work, with the exception of those copied from Soemmering, which form the most valuable part of the work. Since the year 1819, a journal of ophthalmology has existed; it was commenced by Guillié with the assistance of Dupuytren, Alibert, Pariset, Lucas, and Nauche, under the title of the *Bibliotheque Ophthalmologique*.* The plan of the work was not such as the state of ophthalmic surgery in France required; and the execution of it soon degenerated from the arrangement with which it set out. Instead of commencing with a natural description of the various kinds of ophthalmia, and the establishment of a new symptomatology, so much required in that country, it immediately leads the reader to details of practice and contains numerous recommendations of empirically employed medicines. Without prejudice, we may say that no compendium of the diseases of the eye is to be found in the French language, at all calculated for general use. If Delarue had considered, says our author, that the eye can only be examined by the eye, (doss das auge nur durch das auge) one would have thought that he would have given a better account of the symptoms, and pointed out more accurately, the diagnostics of the affections of that organ. What Roux, Lassus, Richerand, and Boyer have said, in their surgical works on the diseases of the eye is, for the most part, very incomplete, and what is to be found in them good, is taken from the works of Wenzel and others. Sabatier's Operative Surgery, the first edition, contains rather a good account of some diseases, but it does not answer the expectations which a perusal of its title-page excites. Monographs on the diseases of the eye, are just as rare in French ophthalmic literature, as they are frequent on other pathological subjects; and if we except Guillié's work on Amaurosis and the Black Cataract,† we know no others. A few inaugural dissertations which have appeared in Paris and Montpellier, need scarcely to be mentioned as exceptions to this observation. The zeal with which the study of physiology has been pursued in France, has brought to light many important points in elucidation of the physiology of the eye. Magendie's investigations on the manner in which objects are represented on the retina, together with the functions of the iris, have been the chief of these; in addition to which, we may mention the mode in which the nutrition of the lens is effected, by Cloquet and Larrey, and the experiments to disprove the existence of the liquor Morgagni by Delarue. It would lead us, however, too far to give an account of all these investigations, which have been of utility to ophthalmic surgery, but which have also been prosecuted by other experimenters. From this brief sketch of the history of the ophthalmic literature in France, it will be seen how little has been done in that country, for the advancement of ophthalmic surgery; and we must, in justice to the German surgeons, acknowledge that it is to them principally

* We give the object of the work in the words of the author. "De reunir une collection des faits, a fin d'en deduire des regles et des principes surs, pour acquerir une connaissance approfondie des maladies des yeux, et de l'administration des medicamens propres a les combattre. Reunir des faits c'est creer une science ou la perfectionner si elle existe."

† *Nouvelles Recherches sur la Cataracte et la Goutte Sereine. Par Guillié. Seconde edit. Paris,* 1818.

that we are indebted for the degree of information we possess on this interesting subject. We have refrained from making any allusions to the state of ophthalmic surgery in this country, or to the causes which have formerly operated to retard its progress. These causes are known, and the measures at present employed will in a short time remove them; on the whole, we may congratulate the surgeons of this country on the industry and attention with which they have of late years applied themselves to this subject; we had at first an idea of introducing a few comparative observations on the state of English, with the German and French surgery, but we find the subject too extensive for the limits of a review, and shall, therefore, leave such a task to be executed by others. We would only observe, that it would prove exceedingly interesting and useful to all parties.

Having arrived thus far, we may take a glance at the ophthalmonosologie and ophthalmotherapie in France. The important subject of inflammation of the eye is very briefly dismissed by the French writers, we should rather say superficially, for although there is a plenty of writing, there is only one monograph on inflammation of the eyes, which is LE FEBURE's. The doctrine of the purulent ophthalmia being contagious, a doctrine which has so much occupied the attention of the German surgeons, has met with no better fate than other equally important inflammations. This is the more extraordinary when we consider how much the French army suffered from its ravages, and how extensively the disease was propagated, so that the French invalid houses were full of persons who had lost their sight. It never attracted any great attention or never produced any accurate description of the disease from the men who had the best opportunities of observing it. LARREY and DESGENETTES only gave their opinions medically on the subject. The works which have appeared from BALTZ in 1815, and from GRAEFE in 1823, are known throughout Europe. GRAEFE has treated, in a masterly manner, the history of the ophthalmia which he believes to be contagious;* he thinks that he has found in some of the Greek classics, particularly in Xenophon's Anabasis, mention made of a similar disease. This is a very splendid work, and has made its appearance in a degree of typographical elegance seldom arrived at. It contains a complete history of the progress of the disease, the symptoms are accurately described, and the treatment is laid down as applicable to each stage of the disease with great precision and certainty. As a monograph, it may be considered one of the most perfect, and must have cost the author much time and labour.† GEORGE BARTISCH, who was chief oculist to Augustus, Prince Elector of Saxony, mentions, in his curious work published at Dresden in the year 1583, entitled Οφθαλμοδουλεια, two kinds of ophthalmia which had a great resemblance to the ophthalmia purulenta, or contagiosa, as it is assumed to be by most German writers of the present day, and the illuminated engravings contained in this biblical curiosity, represent states of the organ very similar to such as are presented in the progress of the purulent or Egyptian ophthalmia. BARTISCH certainly does not mention that this disease propagated itself by contagion, and being unable to explain its rapid spread, he had recourse, in common, with his cotemporaries in all cases of difficulty, to supernatural agencies. He ascribed it to a species of witchcraft, and divides

* Dir epidemisch-contagiöse Augenblennorrhöe Aegyptens in dem europäischen Befreiungsheeres ihre enstchmy, Erkenntniss, vorbungung und Heilart, während des Feldzuges, 1813, 1814, 1815, von. Dr. C. F. Gräefe, 1823.

† At the same time it must be remembered, that the author has had the benefit of the works of RUST and other very respectable authors.

the disease into two kinds, the hot kind of magical influence, the *fascinum, fascinatio calida*, and into the cold kind, or the *fascinatio frigida*, for each of which kind of distemper he has given engravings of the shapes and composition of certain amulets which he found most efficacious in stopping its progress! Of the French, DEMOURS is of opinion that the disease was spread by an epidemic influence, whilst GUILLIÉ and others assert that they are quite satisfied of its being of a contagious nature. He stoutly vindicates the utility of the contagious doctrine, and thinks he has quite set the matter at rest by the following experiment:—With GUERSENT, first physician of the "Hôpital des Enfans Malades" in Paris, he introduced, under the eyelids of four boys, born blind, some mucus taken from the eye of a person labouring under the second stage of purulent ophthalmia; they were removed from all other infection supposed to have a prejudicial influence, and inhabited an airy healthy situation, yet they each had an attack of purulent ophthalmia.

Iritis, its causes, symptoms, and treatment, have been thoroughly investigated, described, and determined by the Germans; and the diagnosis of the complaint established beyond the possibility of a doubt. The appearance and figure of the pupils, which, of themselves, sufficiently characterize the disease, do not meet with that degree of attention from the French surgeons which the importance of the case requires; Dupuytren only excepted. The treatment of iritis is not yet thoroughly understood by the majority of the French surgeons, and many eyes are yearly lost in consequence. The diseases of the anterior chamber of the eye have been well studied by LANGENBECK and WEDERMEYER in Germany, and by WARDROP in England, whilst in France it receives so little attention, that Dr. Ammon assures us he never heard such a thing named as an inflammation of the membrane of the aqueous humour, although that membrane was first demonstrated by BICHAT. The existence of this membrane has been since questioned by RIBES. From the observation of the Germans on this subject, the treatment of hypopion is much improved, they trust rather to the employment of antiphlogistic measures to check the inflammatory action productive of the hypopion, than have recourse to puncture of the cornea so frequently practised by the French, which in its immediate effect is only palliative, and in its remote effect, aggravates the disease. Among the various modes for the removal of opacities of the cornea, that of Dupuytren is the most in vogue, which is nearly as follows:—If inflammation of the cornea or surrounding soft parts exist, bleeding or leeches are first ordered during three or four days, mild purgatives will be given; and as soon as the inflammation is removed, some of the following powder is blown into the eye morning and evening.

| | |
|---|---|
| Tuthié preparée, | } āā parties égales. |
| Sucre candi, | |
| Calomelas Anglais, | |

This practice is continued for several weeks, at the end of which time, it frequently happens that the opacities are removed. If the opacities should be very large or have continued a great length of time, a seton is put into the neck. In cases of important disorganization of the cornea, or when there may be any considerable staphylomatous projection of this part, DELARUE is in the habit of frequently touching it with the *lapis infernalis* and using a collyrium in which some of the *lapis divinus* is dissolved; a complication of treatment, which one might suppose equal to the removal of any ailment ghostly or corporeal. But, in addition to this, he employs pressure upon the distended part, and thinks, in this way, to remove the complaint. Not later than the year 1779, Pellier of Montpellier was in the habit of treating leucoma corneæ by introducing a seton of two threads of silk through the opaque substance of the cornea, which seton was moved daily and besmeared with basilicon ointment. DELARUE has lately imitated PELLIER's example, and assures us that he has performed a similar operation with success; a practice at which Dr. Ammon has expressed his surprise in the words of Juvenal, "non hic cautatur, res vera agitur," a mode of treatment also, to the description of which Delarue has devoted several pages of his work! The ophthalmo-therapie, as it is at present found in Germany, is considered by the author to be better than in any other country of the globe: for this we cannot blame him; it is *ganz natürlich*, but if he had only said that it is better than that employed in France, he would have been right. One thing is certain, the Germans have very few specifica ophthalmica, whilst the French have an abundance, and wherever specifics are much resorted to, there an ignorance of pathology and therupeutics will be found to prevail. The Germans, in their treatment of ophthalmic diseases, puzzle themselves very much about the constitutional diathesis, and make trifling alterations in the treatment according to the prevailing dyscrasy. In an ophthalmic ward, a dialogue of this sort may be frequently heard:—Is this a *blennorrhœa* or *ophthalmia catarrhalis?* It is blennorrhœa, but only *blephacoblennorrhœa.* But is it *blephacoblenorrhœa rheumatica, metastacica* or *strumatica?* Ah, that is a question not easily answered, now let us examine. The examination of all the signs, direct and indirect, then commences, accompanied with half an hour's squabbling between the teacher and pupil, in which we shall leave them, and all this to find out whether the lotion shall be applied warm or cold, or whether the patient shall have cooling or spicy cathartics. The treatment employed is not so strictly antiphlogistic as that in France, and the German surgeons reprobate the French for their too frequent use of the lancet and Sangrado practice, and say, that whilst they use indiscriminately severe general antiphlogistic treatment, they counteract its effect by blowing different kinds of powders into the eye, and using irritating local applications. The process of blowing powders into the eye may be seen daily at the Hotel Dieu, where Dupuytren is a great friend to the practice. This surgeon, after an experience of ten years, as to the use of the belladonna internally, is perfectly satisfied of its utility, in cases of scrophulous ophthalmia, and in cases of inflammation occurring after depression of the cataract, which he considers generally to be retinitis. He gives the belladonna either in the form of powder, or extract, made into pills, a moderate dose of which he gives every two hours. Dupuytren has of late paid particular attention to the formation of encysted tumours of the cornea, and such as form between the lamellæ of the cornea, a subject which has not yet attracted much attention in Germany. Dr. SCHMATZ of Pirna, has spoken very laudatory of the effects of the senega root, in some affections of the eye, which he regards as the vegetable calomel. The use of senega in hypopion has been mentioned by WINDT of Gelangen.* Within the last twenty-six years, this physician had opportunities of trying the effect of this remedy in three cases of suppuration of the eye. We profess that we cannot understand what the peculiar efficacy of senega can be in such cases, and mention it only as a fact, possessing more of what is curious than meritorious. The subject of cataract has received the greatest attention from German surgeons and physicians, and since the time that WALTHER of Bonn first drew their attention to the fact, that opacities of the capsule, and of the lens itself, were frequently owing to inflammatory action, the knowledge of the subject has become more ex-

* Annalen des klinischen Instituts auf der Akademie zu Gelangen, von Dr. Friedrich Wendt. Astes Heft. 1808.

tensively diffused, and this theory is generally adopted; whilst in France, a chaos of opinion still prevails on the subject; by some, the formation of cataract is ascribed to the metastasis of some external irritation, to the sudden suppression of some accustomed discharge, or a disproportion between the quantity which is excreted or absorbed within the capsule. Guillié ascribes the formation of cataract to a process somewhat similar to what takes place in necrosis in bone, and thinks that the softer parts undergo a degree of chemical decomposition, of which the opacity is the tertium quid. The modern French ophthalmic writers, doubt and deny that inflammation is the cause of cataract, and they endeavour to prop up this opinion by arguing that, no inflammation can take place where there are no red blood-vessels: in addition to which, it is said that cataracts occur most frequently at that period of life in which the vascular system is the least active. The formation of matter within the capsule, is a fact sufficient to show the fallacy of any such assertion. The minute distinctions between the different kinds of cataract, as they are taught in the cliniques of Germany, are lost sight of in France, although professedly they are observed, and it was hence no un-uncommon circumstance to hear the surgeons disputing, says Dr. A. whether a cataract was hard or soft. Of late years, the black cataract, and the congenital cataract have principally engaged their attention. Delarue has attempted to prove to demonstration, that the capsule is only opaque in congenital cataract, and this opinion was for some time in vogue, but whether the French surgeons are of this opinion generally or not, they usually defer operating until the child has grown up. Delarue thinks that he has proved the above assertion to be true, from having, in some cases, found, that the light reached the retina when he perforated the capsule through the cornea, forgetting that the lens might in his cases, as usually happens in congenital cataract, have become absorbed. The early known and frequently described black cataract, the cataracta nigra, has found a zealous supporter in Guillié. Delarue thinks that the black star shews itself in countries far north and south of the Æquila. The diagnosis of cataract is not so good as in Germany. The needle operations performed in Paris are principally modifications of Scarpa. S.

---

2. *Obliteration of the Canal of an Incarcerated Intestine.* Dr. Bishopp, of Thornby, has related an instance of this rare occurrence, in the 5th Number (New Series) of the Medical Repository, which we shall notice on more accounts than the pathological one in question. A young man, aged 25, was ruptured, while carrying a sack of corn, on the 27th March. He was seized with the usual symptoms of hernia, and was seen at noon of the same day, four hours after the accident. Dr. B. bled the patient and used the taxis, but without success. Oily and tobacco glysters were then employed, and cold applied externally, He was now left till the following morning, (28th) when he was found *in statu quo*. The venesection was repeated, and carried to syncope, when the taxis was again tried, but with caution. No success. The operation was proposed, but rejected by patient and friends. 29th. Little variation in the symptoms. The taxis again slightly tried. 31st. He now consented (when it was too late) to the operation. The sac was found to contain about three ounces of serous fluid. "The intestine was slightly discoloured, its texture apparently uninjured, but very firmly embraced by the mouth of the sac." It was reduced without difficulty, and the urgent symptoms nearly ceased for some hours, but afterwards returned with increased violence, and death soon closed the scene.

*Dissection.* Marks of inflammation were seen in different parts of the abdomen, but no approach to gangrene. The inflammation was most considerable in that portion of the gut (ileum) which had been strangulated. When this portion was cut out, it was found that the canal was completely obliterated by adhesion of its mucous surfaces. It was shewn to Mr. Brodie, who corroborated the statement above made.

A similar instance is related by M. Ritsh, in the Memoirs of the Academy of Surgery, volume four. We shall state a few of the particulars.

A man, 45 years of age, had had an inguinal hernia for several years, but which was kept up by a bandage. One day, while lifting a heavy burthen, the hernia protruded, and symptoms of strangulation quickly ensued. The taxis having failed, M. Ritsh was called in, and performed the operation. The intestine was found inflamed, but not so much so as to contra-indicate reduction. The symptoms of strangulation were calmed. But no stools came away, the symptoms were renewed, and the patient died twelve hours after the operation. On dissection, the ileum was found much contracted in two places where it had passed the ring. These portions had their parietes completely glued together by adhesive inflammation, so that the passage was entirely obliterated. This was the cause of death.

In the first place, we may remark that, in many cases—perhaps in most that fail after the operation, death does not take place from the inflammation or mortification of the strangulated portion of gut, but from non-restoration of function in the part so strangulated. The function or the passage is probably often destroyed by the throwing out of coagulable lymph and the consequent adhesive inflammation. Every body knows, what indeed was pointed out long ago by John Hunter, that the mucous membranes will occasionally adhere by the same process which agglutinates the serous structures. Mr. Geoghigan, of Dublin, in a letter to John Abernethy, (Ed. Journal, Jan. 1824,) affirms that the reduction of hernia, whether by taxis or operation, under an inflamed and tumefied state of the prolapsed gut, frequently fails of success, in consequence of its being in an impervious state, arising from the establishment of an agglutination of its villous coat. We regret, with Dr. Bishopp, that Mr. G. has not supported his statements by the details of *post-mortem* examinations, though we have no doubt of the fact, from the cases now quoted, and from what we have seen ourselves.

In Gräefe and Walther's Journal, there is a case related by Dr, Guntha of Cologne, where a patient died after reduction of a strangulated hernia by the taxis. On dissection, a portion of small intestine, about an inch and a half in length, was found so constricted, that its canal was almost entirely obliterated. The viscera of the abdomen generally were in a healthy state.

We have entered thus far into the subject, chiefly to draw the attention of British Surgeons to a more early recourse to the knife than they seem inclined to. It is abundantly evident that both of the first two cases mentioned above, were lost for want of an early operation. After bleeding to syncope, and using the most approved means of taxis for a *few hours*, without success, we maintain that all further delay of the operation, and all subsequent efforts at reduction are made at the risk of the patient's life and the surgeon's reputation. In subsequent attempts by the taxis we may, indeed, succeed in pushing up the intestine from its prison in the sac, but it will often be done when the incarcerated portion has lost the power of carrying on its function. We need hardly say that the same observation applies to a late operation. If it is to be done, the sooner the better. We should take a lesson from some of the Continental surgeons, especially M. Dupuytren, who seldom or never wastes more time than is taken up with the first attempt at reduction. The second attempt is with the knife—and his success is in proportion to this early recourse to an operation.

---

3. *Artificial Anus.* Dr. Martland, of Blackburn, has lately stated an interesting case of the melancholy alternative between death and a loathsome substitute for a natural passage.

A man, 44 years of age, became affected with difficulty in voiding his fæces, which at last amounted to a complete obstruction. Bougies could not be passed upwards, and purgatives of the most drastic kind had no other effect than that of rendering the distention of the abdomen greater and more distressing. Under these circumstances, nothing remained but an operation to save life.

In the presence of Messrs. Barlow, Bailly, and Cort, Dr. Martland made an incision in the left iliac region, from near the anterior superior spinous process of the ilium, downwards and inwards for the space of about four inches. The muscles were then cut through to the same extent, and the peritoneum laid bare. This was cautiously opened by a bistoury, when the colon presented itself, known by its longitudinal bands. An opening of an inch and a half was made into this gut, having first secured it, by two sutures, to the two extremities of the wound. A large quantity of liquid fæces and air immediately escaped. Two other ligatures were now inserted, in order to keep the opening in the gut in correspondence with that in the integuments. An attempt was made to pass an œsophagus tube from the wound down to the anus, but in vain. An artificial anus was thus established near the groin, which still continues, after a lapse of many months, and there is no prospect of the original passage being opened. After various contrivances, the patient has ascertained that many folds of cotton, over which is a kind of pessary, and then a bandage, is the best apparatus for restraining the fæcal matters till he has an opportunity of discharging them by the artificial outlet.

Life is sweet, and Nature strongly abhors the King of Terrors—therefore, the exertions of the surgeon in thus prolonging existence, even with such a terrible sacrifice, are praiseworthy.

4. *Early removal of Cataract by Absorption.** In a hasty view which we took of Mr. Stevenson's work, in a former Number of this Journal, our limits did not permit us to fully notice the fifth chapter, " on the advantages resulting from the removal of the different species of cataract, at an early period after their formation, by the absorbent practice." We think the views contained in this chapter are worthy of being more generally known, and on this account we shall introduce an analysis of the chapter above-mentioned in this place.

Mr. S. observes that the physical means for removing cataract, have hitherto been reserved for the ulterior stage of the disease, when the sight has become greatly impaired or wholly lost. He, therefore, claims the priority of proposing an operation, " without regard to the kind or maturity of the cataract," so soon, in short, as its character is sufficiently disclosed to enable us to decide upon the real nature of the disease. The plan here recommended aims at the *prevention* of actual blindness, by taking advantage of the absorbent powers, *before* the lens or its capsule has become so far disorganized as to occasion more than an imperfect eclipse of vision. The leading principle of the practice is by no means new—and it is not a little singular that Celsus himself describes in less than two lines, the modern operation of cutting up the lens for solution and absorption. " Si subinde redit eadem (cataracta) acu magis concidenda, et in plures partes disrepanda est." Lib. vii. In the other works of Bannister, Read, and others of our own country, there are evidences of the effect of absorption; but we shall not touch on the modern and living authors who have written on this subject. Mr. S. makes observations on them severally, and remarks, p. 150, that, from due consideration and ample experience, he is led to the belief, that couching or extraction, excepting under very peculiar circumstances, can only be necessary or proper in the *indurated* state of the lens—and that the removal of the several kinds of *soft*, *fluid*, and *capsular cataract*, is more easily and safely accomplished by division or laceration, with the subsequent process of absorption. The main point in this operation is to comminute, with a proper needle, the opake crystalline, or tear its capsule, when morbidly affected, in order that they may, in these states, more readily undergo the process of solution and absorption; whilst, at the same time, the smallness of their size tends to obviate pressure, and consequent irritation of the adjacent parts. The translation of these disorganized fragments into the anterior chamber is of greater importance as respects the prevention of irritation, which would be apt to result from the pressure of the lens on the iris, if detained in the posterior chamber, than as regards the acceleration of absorption. " So long, then, as the morbid crystalline continues soft, or its capsule is easily lacerable, a cataract of either description may readily and safely be reduced, by the agency of the needle, to a state fit to be acted on by the absorbents." Now the changes which disqualify for this the most preferable mode of cure, are the effect of age, or of allowing the cataracts to remain unmolested until they have undergone those unfavourable transmutations—" a period of time which varies from a few weeks to some years." It is to be remembered, that although old cataracts are sometimes found soft, the process from hard to soft never takes place. The disease is assailable by the needle whilst recent, whether it be of a simple or compound character—whether it consist of an opacity of the lens or capsule only, or of both—whether it be congenital, second-

* Mr. Stevenson, Chap. v. of his work on the Nature, Symptoms, and Cure of Cataract.

ary, or even accidental. By the agency of this instrument, the surgeon is enabled to operate upon each respective modification of cataract in its early stage, so as to subject the disorganized and divided parts to the process of absorption. Another argument in favour of this process is, that it may be effected not only with the most striking degree of present pain, but likewise with little comparative risk of subsequent inflammation. Such are the reasons for an early operation, and our author thinks it very remarkable, that they have not been sooner appreciated. The treatment of cataract, he observes, has been, for many ages, and still continues to be, conducted upon principles, the reverse of those which regulate our conduct in the management of every other disease. "What," says he, "would be thought of a surgeon were he to propose waiting until an *occult* has become an open cancer, before he had recourse to the knife for its extirpation? Or what opinion would be entertained of the abilities and qualifications of a physician, who should venture to inculcate the propriety of withholding his remedies in different kinds of fever, until the symptoms had reached their acmé, or attained their full force and malignancy?" It is needless to go with our author into a refutation of the absurd doctrines of the ancients respecting the supposed necessity of *ripeness* in the cataract before any operation could be safely performed for its removal; nor are we inclined to stand judge between our author and modern surgeons who still advocate the propriety of deferring an operation till sight is lost—our object in this short article being merely to make known the peculiar views of Mr. Stevenson. For the arguments which he has adduced in favour of his own mode of practice, and in answer to the objections made to it, we must refer to the chapter abovementioned; concluding with the following corollaries, which appear to our author as fairly deducible from the premises set forth.

"1*st*. That the mental distress, the occurrence of blindness, and the contingent difficulties and dangers which may arise from waiting an indefinite period for the lens to become *ripe*, or in a state fit for the common operations of couching or extraction—(a change in its texture which, when it does occur, is often exceedingly tardy, the period of which cannot with certainty be anticipated, and may never arrive)—are wholly obviated by the plan proposed.

"2*dly*, That the mode of operating recommended, if had recourse to as soon as convenient after the formation of the disease, is not only applicable to every variety and complexion of cataract, but is likewise attended with a very inconsiderable share of pain to the patient, and with little comparative difficulty to an experienced surgeon.

"3*dly*, That a repetition of the operation will be rarely called for, if the process be, in the first instance, properly executed, in consequence of the absorptive process going on with great rapidity, whilst the crystalline remains *soft*, or its capsule is easily lacerable; and, at the same time, there will be much less disposition to subsequent inflammatory irritation, than after either form of cataract has acquired a greater degree of consistence or tenacity.

"4*thly*, That the needle, under these circumstances, can be employed to break up the slightly opaque lens, or reduce its capsule to shreds, with the smallest imaginable injury to the eye.

"5*thly*, That in consequence of the above advantages, and from the very nature of the process, vision is rendered at least equally good as after the most fortunate cures achieved by the operation of couching or extraction, at the same time that the possibility of a *secondary* cataract is effectually obviated.

"*6thly*, That should not the incipient disease in the less affected eye spontaneously disappear, the restoration of sight in the one operated on will generally take place before the opacity in the former has made such progress as materially to interfere with its function.

"*7thly*, That, consequently, by this method of proceeding, the patient is guarded against the occurrence of blindness instead of being kept in a state of anxiety and suspense, in constant expectation of its arrival, and by means which, at the same time that they are nearly destitute of pain and hazard, have a direct tendency to restore to the organ the greatest degree of perfection of which it is susceptible.

"*Lastly*, That the plan of treatment suggested, whilst it challenges all the advantages which the customary and old methods of operating seek to afford, is decidedly preferable to both, for the reasons already assigned; and in being not only easier of execution, much more certain in its effects, and comparatively free from danger, but also in its universal applicability to every description of cataract at the early period of its formation, when neither of the common processes can be resorted to with safety or success." 234.

---

5. *Hydrocephalus tapped.* We do not conceive that a cure will ever be found for hydrocephalus (whether the fluid be in the ventricles or between the membranes of the brain) in the operation of tapping. But it is our duty to prolong life, even where existence is a misery and where death would be a blessing! A good many operations have now been performed for the removal of water from the brain, and few, if any of them appear to have done harm, though none of them have been ultimately successful. Mr. Sym, of Kilmarnock, has recently published a case of "congenital" hydrocephalus, where the child's head "was of an uncommonly *diminutive* size" at birth. For six weeks the infant was very fretful, yet he increased in bulk, sucked with avidity, and exhibited no symptoms of disease. At the period abovementioned, however, it was observed that the head became tumid at the fontanelles, and he began to waste in flesh. At the age of eleven weeks, Mr. S. saw the child, when the head had attained a considerable size, and, when placed between the eye and a strong light, was diaphanous down to the temporal muscles. Medicines were prescribed, but had no effect; and the integuments covering the wide interstices between the bones becoming very thin, Mr. Sym scratched an opening, with a lancet, in the posterior fontanelle, whence there issued six ounces of a salt-tasted serum, leaving the head so flaccid, that it was found difficult to approximate the bones. The child suffered no inconvenience from the sudden removal of the fluid. He revived after the operation, slept well the succeeding night, and took the breast more greedily than for some weeks previously. The water soon accumulated, and the operation was frequently repeated afterwards. These temporary mitigations did not prevent death at the age of six months.

*Dissection*. The dura mater adhered firmly to the bones of the skull, "and the thickened arachnoid membrane formed a large sac, containing 2½ pounds of limpid serum, and partially divided into two chambers by the falx." The cerebellum and pons varolii were natural, as were also the nerves arising within the cranium; "but, in place of the cerebrum, there was only a small, firm, flat mass, not larger than a garden bean, lying over the cella tunica (turcica.) It was so much compressed that it was quite impossible to recognise in it any part corresponding to the structure of a healthy brain."—*Ed. Jour.*

This is a more interesting case, in a phrenological point of view, than Mr. Sym seems to be aware of. We regret that he has made no remarks on the comparative development of the mental functions in a child of six months old, with a cerebrum only the size of a garden-bean. We hope the phrenologists of the North will brush up Mr. Sym's memory on this point.

---

6. *Amputation.** When lawyers shall cease to wrangle at the bar, and to contend for different interpretations of the law, then shall doctors agree as to the nature and treatment of diseases, and surgeons as to the best modes of operating in each particular case. When this millennium arrives, strange effects will follow among the professors of physic! Their brains will become as diffluent as water, for want of thought, in the same way that muscles become pale and flabby for want of exercise. Medical societies will cease to exist, for what can their orators have to say? Medical books and journals will then roll no more from the press—the critic's cranium will be as empty as Yorick's—and the gall in his ink-stand as dry as portable soup! But, since such an æra is not yet at hand, we must record the discrepancies of the profession, as collisions of intellect, which often emit sparks of useful light.

One would suppose that if any operation in surgery was settled on a firm basis, it would be that of amputation. But this is not the case. The French surgeons advocate granulation—the English, adhesion by the first intention. Some of the latter prefer the flap, and some the circular incision. Mr. Dewar's design, in this paper, is to offer a few remarks on the comparative merits of these last modes of amputating. The primary objects, he thinks, of the operator must be, celerity, union by the first intention, and a *good stump.* The last is, unquestionably, the great desideratum. If the bone be not covered with a good mass of muscle and integument, the patient cannot expect the comfort and convenience of an artificial leg. Although we have seen and fashioned many a good stump by the circular incision, yet we are free to confess that, unless much care is taken, the muscles will contract irregularly, adhesion by the first intention will be prevented, and a very inadequate cushion will cover the face of the bone. These are inconveniences which all must have seen and experienced, though we think they are considerably exaggerated by Mr. Dewar. We are friendly, therefore, to the flap operation, where it can be performed, as it may in most cases. Different modes of effecting the flap have been recommended. The following is Mr. Lizars' plan, as given in the sixth part of his description of anatomical plates.

---

* Mr. Dewar. Ed. Journal, October 1825.

" The patient being placed on a table, with the leg hanging over, and supported by an assistant, and the roller or pad, with the screw of the tourniquet, placed over the artery, and sufficiently tight to compress the circulation; and the skin being retracted by an assistant, two semi-elliptical incisions are to be made with a large scalpel through the skin and contiguous muscles, from the patellar to the popliteal aspect, as deep as possible, with their convexities large, and pointing distad, and their extremities meeting patellad and poplitead. A repetition of each incision, boldly made, enables the operator to reach the bone, which is to be cleaned of the adhering muscular fibre; the flaps are then retracted, and the bone sawn, by long gentle sweeps of the saw." 267.

Mr. Dewar practises the operation in this manner:—The patient being laid in the usual posture, one assistant supports the limb, while another compresses with his thumb, the femoral artery passing out of the pelvis. The operator then grasps the whole of the flesh on the outside of the thigh with his left hand, and transfixes it close by the bone with his long knife, cutting his way out, and leaving a flap of the length he judges necessary. The other half of the thigh is cut in a similar manner, from within outwards, in the shape of another flap. The knife is then passed round the bone to divide any muscular fibres which may adhere to it. The flaps are held asunder while the bone is sawn, and the vessels are then secured. This operation, our author avers, does not require more than a minute for its performance—a celerity to which we have no objection, where there is nothing endangered by it. The wound consists of two flaps of equal size and length, which are easily retained in contact by adhesive plaster or stitches. The parts composing the stump, all retain their natural relations to one another—the vitality of the skin is unimpaired, as is its connexions with the subjacent muscles—tissues similar in structure and function are brought together when the wound is closed, " and union by the first intention is not merely a probable, but a general (occurrence) recurrence." Four cases are related by Mr. Dewar, in illustration of the good effects resulting from this mode of amputation, which we think is superior to the old circular incision—and particularly to that of Dupuytren, who cuts down to the bone with one fell sweep, without regard to skin, muscle, or cellular membrane.

---

7. *Rupture of the Lig. Patellæ.* In the year 1817, a captain of a merchantman was thrown forward on the deck of his vessel, in a storm, and in the attempt to save himself from falling, ruptured the ligamentum patellæ, merely by the force of the extensor muscles, for the knee did not come in contact with any hard body. They had no surgeon on board, and the knee-pan " was doctored the best way they could." In the course of seven weeks it broke three times from the situation to which they had brought it by bandages. When he came within the reach of a surgeon the case was beyond the reach of surgery. " One surgeon recommended him to have the part opened, and inflammation excited in the ruptured part." To this the patient wisely objected. The motion of the knee was at first very imperfect, but by degrees it arrived at such a degree of strength and motion, that the captain can now move the limb with ease and freedom in every direction, rising up and sitting down without the slightest difficulty. When examined by Mr. Thompson, of Whitehaven, eight years after the accident, he found that the patella had been forcibly torn from its attachment to the tubercle of the tibia, and removed, by the contraction of the muscles, five inches from its original situation. No part of the bone had been broken —the tendon alone had been ruptured. The patella remains permanently at the distance of five inches from its original site, except when the extensor muscles are in strong action, when it ascends about three quarters of an inch higher. The lower edge of the bone does not appear to have any attachment to the parts in the neighbourhood; but on its sides it seems fixed to the muscles by a rather loose hold, which is more particularly perceived when the patella is extended. A dragging is then observed affecting the inner edges of the vasti. Those parts of the vasti muscles inserted into the aponeurosis leading to the head of the tibia, would not appear to have partaken at all of the injury, and it is probably owing to this circumstance that the free motion of the joint is kept up. The thigh appears wasted; but the leg is not altered in appearance.—*Med. Repos. September* 1825.

---

8. *Closure of the Pupil in Iritis.* [Mr. Mackenzie, Med. Journal, No. 318.] No explanation, Mr. M. says, has ever yet been given of closure of the pupil, though it is the most important symptom in iritis. Inflammation of the iris seems, to our author, an inadequate cause—the effusion into the posterior chamber might prevent the opening of the pupil after being closed, but does not account for the first closure. Intolerance of light is seldom an attendant on iritis, and, therefore, cannot be the cause. Inflammation of the retina is not the cause, for this part is often sound while the pupil is completely closed, so that vision may afterwards be restored by the formation of an artificial pupil. And now, having seen, or rather supposed, what are *not* the causes of the pupillary contraction, our author proceeds to develope his ideas of the phenomenon. Lest we should misrepresent his meaning, he shall speak for himself.

" In the Lectures on the Structure and Diseases of the Eye, which I have given for the last seven years, I have ventured to ascribe the closure of the pupil in iritis to the fact, that the natural state of that opening (the state into which it falls during the insensibility of sleep,) is closure, not expansion. The discovery of this fact we owe to Fontana;* its truth has been acknowleged by Janin† and Cuvier,‡ and may easily be ascertained by any inquirer. Approach a child fast

" * *Dei Moti dell' Iride.* Lucca, 1765; page 21."

" † *Mémoires sur l'Œil.* Lyon, 1772; page 8."

" ‡ *Leçons d'Anatomie Comparée,* tome ii. page 409."

asleep, and suddenly raise the upper eyelid; the pupil will appear all but closed, and only gradually, as the state of sleep is broken, will it be seen first of all to expand, and at last to accomodate itself to the degree of light to which the eye is exposed.

"Iritis is always aggravated, and often appears to commence, during the night, particularly after the operations for cataract. The probable cause of this is the contraction of the pupil, or rather the expansion of the iris, during sleep. Hence the propriety of applying the extract of belladonna to the forehead every evening, particularly in cases of incipient iritis.

"If no means are used to counteract the progress of iritis, the pupil is observed to become, day after day, smaller and smaller, and less and less dilatable, till it be completely closed and bound down by effused fibrin to the anterior hemisphere of the crystalline capsule. If belladonna only be employed, without the detraction of blood and the exhibition of mercury, the pupil will be kept dilated; and, if the narcotic has been applied early in the disease, the pupil will be widely expanded, and in this state will become adherent to the crystalline capsule. The detraction of blood, to lower the sanguiferous action,—the use of mercury, to prevent the effusion of fibrin, or to bring about its absorption, if already effused,—and the application of belladonna, to counteract the return of the iris to its natural state of rest, relaxation, and expansion, during sleep, makes up, then, the chief parts of the treatment of iritis, whether the disease be traumatic, atmospheric, or syphilitic; and the belladonna ought to be applied, not irregularly or merely every second or third day, but regularly every evening.

"*Spreull's-court, Glasgow; June* 1*st*, 1825."

We know Mr. Mackenzie to be as clever a little fellow as any within the walls of Glasgow; but we cannot help thinking the foregoing explanation a piece of refinement far too finely spun. If the pupils be closed, or nearly so, in sleep, (and the same may be said of the eyelids also) it only proves that closure is their natural state *during sleep;* and how this can be connected with iritic closure we cannot conceive, unless it be maintained that iritis always takes place in sleep, which would be rather strange, since the disease is no great friend to repose. To us, the cause of the pupillary closure appears to be simply *irritation.* Let the eye, in health, be exposed to strong light, or any other source of irritation, and the pupil becomes contracted. Why, then, should not a deep-seated inflammation produce the same effect? Mr. Mackenzie thinks this inflammation an inadequate cause, yet he offers no reason for so thinking. But whether the pupillary contraction be natural or morbid, it appears to aggravate the iritis, and, therefore, the application of belladonna is useful as an auxiliary in the treatment of the disease.

---

9. *Cranial Fracture,—Depression, &c.** A young lad, nine years

---

* Dr. Corban. Med. and Phys. Journal, March 1825.

of age, received a kick from a horse and was reported to be just expiring. Dr. C. arrived two hours after the accident, and found the boy quite cold, more especially the lower extremities—face pale—pulse feeble and slow—breathing laborious—pupils dilated, but sensible to light—stomach irritable. Much blood had been lost from the wound on the forehead. Cordials, in minute portions, were exhibited, and the feet put into warm water. The frontal bone was found denuded to the extent of three inches, presenting a fracture and deep depression of bone, beaten in with such violence as to rupture the dura mater, through which a dessert spoonful of medullary matter had escaped. The boy soon became rational, after the stunning of the blow had subsided, and answered questions distinctly. Dr. C. cleaned the wound, applied adhesive straps to keep the lips of the wound together, and administered proper medicines internally. Passed a bad night, being somewhat delirious. *Second day.* Pulse 110, full and strong—tenderness in the region of the liver. Bled to 18 ounces—purgatives. *Third day.* Delirious during the night, but sensible this morning—bowels free—suppuration in the wound—a portion of brain in the wound—pulse 108. *Fourth day.* Stomach rejects every thing—lips of the wound tumid and gaping—sanious discharge, mixed with medullary matter—severe head-ache—pulse 115, and sharp. Temporal arteriotomy to 12 ounces—poultice to the wound. *Fifth day.* Slept last night—feels better to-day—bowels open—stomach retentive—abundant discharge from the wound, and of a better quality. With some variation of symptoms, requiring blister—cathartics—but no farther detraction of blood, the boy recovered. There was a deep depression of skull left.

Dr. C. advances this case in favour of the "*Medecine Expectante*;" but wishes to be clearly understood as considering the trephine "to be a very valuable resource, where symptoms of compression, or internal accumulations of matter exist, attended with stupor." We cannot do better than refer to the excellent paper of Mr. Wise, in a late number, for information on this nice point of surgery.

---

10. *Peculiar Ulcerous Affection.** This was a creeping ulcer of many years standing, which began on the back of the head, and crept down along the neck and back, healing behind as it advanced, and never occupying a large space at any one time. The surface of the sore was found to be extremely irregular, with hard, rugged, and elevated edges, assuming very much the appearance of *noli me tangere.* The deepest parts of the surface shewed granulations, secreting pus of pretty healthy appearance. The more elevated portions seemed to be the cellular substance which had undergone a peculiar change, assuming very much the appearance of *well macerated ligament*, being tough, insensible, and fibrous in its structure. Various means were tried to stop the progress of this ulcer, but nothing succeeded till the sound integu-

* Mr. James Braid. Ed. Journal, January 1825.

ments in its front were destroyed by means of caustic. This process insulated, as it were, the ulceration, and completely arrested its farther advances.

---

11. *Injuries of the Head.* The remarks of our intelligent correspondent on the neglect of the trephine, are well illustrated by a case related at page 347 of the London Medical Repository for April last.

"The patient was a boy, nine years and a half old. On Monday, the 7th of October, 1807, he had received a severe injury on the head. His father, in a fit of passion, threw a small poker, not more than half an inch in diameter, at his wife, which missing her, hit the head of the child, and penetrated the skull, so that it remained upright, till drawn out. On examining with a probe a short time after, I readily discovered a depression of bone, but merely by the probe catching against the edge of the superior part of the bone. The child at the time exhibited no severe symptoms; and, with little interruption, continued to improve till the following Sunday, when he died. On the Wednesday there was a slight paralysis of the right leg, the opposite side to the injury, which, however, gradually went off, and on the *Sunday he appeared convalescent, 'was running about the ward, and seemed quite rejoiced that his leg was quite recovered.'* 'Soon after dinner, he was suddenly seized with sickness and vomiting,' and quickly became again paralytic, appeared at seven o'clock in the evening perfectly moribund, but survived till two o'clock the next morning.

"'*Dissection.* Discovered a perforation, situated about half an inch above the squamous portion of the temporal bone, in the left parietal bone, and about a quarter of an inch from the coronal suture. The opening on the outer surface was completely circular; but, upon sawing through and removing the skull-cap, the inner table of skull was found driven in, in three or four separate portions, and pressing upon the brain. There was also a considerable deposit of coagulum of blood on the dura mater, for several inches round the opening through which the instrument had passed into the brain. Removal of the dura mater exhibited the surface of the brain of a brown dusky hue, apparently from diffusion of blood, and, at the point of injury, a slight suppurative process. Perpendicularly slicing the brain exposed the passage of the weapon through the substance of it even into the thalamus, where the injury seemed to terminate. The brain, in the course of the injury, was soft and pulpy, and diffused with blood.' In answer to some inquiries which I made respecting the particular nature and extent of the injury, I was informed, 'that the point of the weapon entered into the substance of the thalamus about a quarter of an inch. The texture of the brain in the vicinity of the injury carried the appearance of brain partly softened by putrefaction, and irregularly red and of a dusky brown hue, but nothing bearing the appearance of pus, excepting at the external opening.'" 347.

In the above case it will be seen how dangerous it is to trust to the existing symptoms, in cases of fracture, with depression of the skull.

Here were no bad symptoms for some days, although fragments of bone were driven into the brain. According to modern rules, no operation is to be performed till bad symptoms occur—when it will generally be too late.

---

12. *Varicose Veins.** Dr. Briquet has denominated this affection phlebectasis, from φλεϐς *vena*, and εχ]ασις *dilatatio*. He thinks the term *varix* inexact, inasmuch as it merely relates to the simple appearance of the malady, and indicates nothing with respect to its anatomical character. Perhaps, however, it would be as well if all our names indicated the simple *appearances* of disease, and did not prejudge or suppose any thing respecting their *nature*. The opinions of men are constantly varying as to the pathology of diseases, but not as to their appearances.

Our author defines this affection to be "a permanent dilatation of veins," presenting a knotty, tortuous, or straight appearance, of blueish colour, increased by muscular exertion and vertical position—diminished in volume or entirely disappearing, on pressure or horizontal posture. Such are the true varices. Another variety is termed the varicose tumour—a third, varicose swelling, not differing materially from the last—and a fourth variety, is simply a development of the small veins, which form a network on the surface of the skin, and are familiar in the face and other parts of the body.

The pathological condition of varicose veins is examined by our author under several heads, and with considerable minuteness. We must endeavour to follow our author through his divisions.

1. *Simple enlargement of Veins.* In this state, the calibre of the vessel is increased—sometimes being distended with blood, and at others, being flattened and not turgid. These veins, when cut across, in the living body, have the power of contracting, as several surgeons, and especially M. Beclard, have proved during operations. The coats of veins in this state, appear rather more condensed than natural, but there is no alteration in their valves. This varicose condition is principally seen in the subcutaneous veins, especially those surrounding indolent swellings—and in the lower extremities of old people, particularly in the vena saphena. It has, also, been observed in the veins of interior parts, as the cava inferior, in cases of induration and enlargement of the liver—in the vena azygos—the veins of the ovaria—the jugulars, in persons affected with asthmatic complaints, difficulty of breathing, or organic diseases of the heart. The veins thus simply enlarged, do not become tortuous, and, in fact, this condition does not deserve the name of *varix*.

2. *Uniform Dilatation, with thickening of Coats.* This state is prin-

* Memoire sur la Phlebectasie, ou Dilatation Variqueuse des Veines. Par M. Briquet, M. D. Archives Générales, Fevrier et Mars, 1825.

cipally observed in the trunk, rarely in the branches of the saphena vein. The venous trunk then forms a uniform cylindrical cord, about the size of a goose quill, from the top of the thigh to the lower part of the leg, nearly straight, or at least with very few sinuosities. The calibre of the vessel is increased—and the lumen, or bore, remains open when it is cut across. Its parietes are thickened, consolidated, and resembling those of an artery. Its internal surface presents longitudinal folds or wrinkles, very regular and distinct, and evidently formed by the inner coat of the vessel, which coat, however, is not thickened in itself—the hypertrophy is seated in the middle coat of the vein.

3. *Unequal Dilatation.* In this species there is sometimes a thickening, sometimes a dilatation without thickening, of the parietes of the vessel. It affects, in preference, the trunk of the saphena, at the bend of the knee, in the leg, and the principal branches of the saphena in the leg. In this species the vein is seen swelled and contracted alternately and irregularly. The dilated parts are sometimes cylindrical—sometimes in the form of bags. This affection, in our author's experience, takes place in the right extremity more frequently than the left.

4. *Predisposition.* There are individuals who shew a great development of the venous system generally—and this happens even in young people. The greater number, however, of varices in the trunk of the saphena take place in muscular people, of tall stature, active habits, energetic circulation, and hæmorrhagic disposition. The complaint is never seen under the age of puberty. It is most frequently observed between the age of 30 and 40. In very old people it rarely takes place, though in them the veins of the lower extremities are naturally large. In respect to sex, it is found much more frequently in the male than in the female. Trades and professions exercise a considerable influence in the production of phlebectasia—especially those of a laborious nature, and where the perpendicular posture is much sustained—as among soldiers, press-men, porters, &c.

5. *Efficient Causes.* Authors have generally considered—1st, The pressure of the column of blood against the parietes of the vein, as one of the efficient causes of its varicose state.—2dly, Those circumstances which diminish the power of resistance in the coats of the vessels.—3dly, Obstructions to the return of blood from the vessel, as abdominal tumours, obesity, utero-gestation, ligatures, &c. Our author thinks that a cause, which is scarcely noticed by writers, and which is yet more efficient than any of the preceding, consists in the active accumulation of a greater quantity of blood than natural in the subcutaneous veins. Thence depend the dilatation of veins around scirrhous and other tumours—the varices which succeed the cessation or suppression of the menses or other periodical discharges—or which appear after an inflammatory affection. It is under similar influence that the vessels enlarge and become tortuous in the neighbourhood of organs pouring forth abundant secretions, as the mammæ during lactation, &c.—the peritoneum and other parietes of cavities, the seat of dropsy, &c. This view of the subject discards the idea of *debility* of the enlarged vessels—on the contrary, it supposes them to be in a state of *greater activity than natural.* In support of this opinion (which, however, was the opinion of Beclard and Chaussier, if not of Bordeu himself,) our author offers many reasonings, which it is not deemed necessary here to quote. There are two cases, however, related—one by Chaussier, and one by the author himself, which are curious, and of which we shall here state some of the particulars.

*Case by M. Chaussier.* A cook became pregnant from time to time. Each time she was informed of the circumstance, towards the second month, by a varicose state of the veins in her legs. She compressed these veins by a bandage, and each time abortion quickly followed.

*Case by M. Briquet.* An unmarried woman, aged 53 years, had varices of the left leg, which began between the age of 15 and 16, at which time the menses first appeared sparingly, and shortly afterwards stopped. From this period, there formed, once a month, in the tract of the saphena vein, a blueish vesicle, which burst and discharged blood by a small opening for four or five days—sometimes in considerable quantities. Then the discharge would cease, and the aperture heal. This continued regularly for six years. The saphena opened in various places, and the cicatrices are still to be seen.

6. *March and Development.* The dilatation sometimes commences in the branches, sometimes in the trunk of the saphena. The vessels first swell uniformly—then present a knotty appearance, the neighbouring parts being increased in temperature, and the circulation in activity. Repose and the horizontal posture disperse these phenomena, but they return after exertion. By repetitions, and in process of time, the vessels become larger, more tortuous, and roll under the finger. Phlebectasia is then established. When in a moderate degree, this affection is merely a deformity, and can scarcely be called an inconvenience; but when more considerable, it causes numbness in the limbs and a difficulty in walking.

7. *State of the Blood in Varices.* We find the blood in varicose veins very similar to arterial blood. When the vein is cut, the jet is often very strong—sometimes *per saltum*, as from an artery, and the flow of blood is always profuse. These circumstances induce our author to think that the capillary vessels between the arteries and veins, in phlebectasia, are dilated, and, consequently, that the communication is more free than in ordinary.

8. *State of the Varicose Limb.* When the disease is of long standing and considerable in degree, the neighbouring cellular tissue becomes indurated—and the limb swells towards night, with some degree of heat and pain. If the person be exposed to much labour or fatigue, the limb gets enlarged, the skin thickened, the cellular membrane indurated, the temperature of the member reduced—and a tendency to ulceration with the least wound.

9. *Supervening Accidents.* Phlebitis often takes place in varicose veins, especially after much exertion, or where ulcers have formed in limbs of this kind. When varicose veins become inflamed, they get larger and harder than before, and the contained blood appears coagulated. The pain is augmented by the least pressure, but there is seldom any redness of the skin or fever. The inflammation generally lasts only a few days, and ends by resolution. The coagulated blood becomes dissolved. When the inflammation spreads, however, to the surrounding cellular membrane, there is an accompanying erysipelas, and it often ends in suppurations and depôts of matter.

10. *Perforation of Varicose Veins.* This happens sometimes, more especially in pregnant women. The vein generally opens, near the ankle, by an almost imperceptible orifice, and pours forth blood abundantly. The least degree of pressure is sufficient to arrest the hæmorrhage. Sometimes the varicose veins burst suddenly, and the hæmorrhage is then profuse. Ulceration is no uncommon supervening accident.

11. *Termination.* The varicose state of veins always goes on increasing if the patient continues to work, and if no means be taken to cure the disease. As old age advances, and as repose is necessarily indulged in, the varicose veins diminish, and often become entirely obliterated. In adult age, the complaint is sometimes cured by the supervention of phlebitis. On the other hand, phlebectasia may gradually destroy the use of the limb, and, by a suite of consequences, bring the patient to an untimely grave.

12. *Treatment.* The ancients used various remedial means. Hippocrates pricked the varices—Celsus recommends them to be cauterized, or removed by a cutting instrument. Ætius also speaks of cauterization and of the ligature. Some have prescribed pressure, but the greater number of the older surgeons speak of excision in such cases. Modern surgeons seem to adopt a palliative treatment in the majority of cases, namely, by bandaging. But it is evident that this measure is very inefficient, and requires more care and skill in its adaptation than fall to the lot of patients. On this account, the ligature and excision have been practised of late by some of our most distinguished surgeons.

13. *Ligature.* This process, employed by the ancients, had been abandoned, when Home, Travers, Physick, Beclard, and others, revived it in England, America, and France. M. Beclard has had but three instances of a fatal termination after this operation in eight years. The object of this operation is the obliteration of the vein, while the current of the circulation is established through another channel—the neighbouring vessels. Mr. Travers thinks that this obliteration is effected without inflammation of the vessels, but this is doubted by M. Briquet. In 31 operations by the ligature, which our author has witnessed, the thread never came away before the 6th day, nor remained longer than the 22nd day. The usual time of separation is between the 7th and 10th days. He does not know how long it requires for the absorption of the coagulum below the ligature. In a few cases, a partial return of the varicose state takes place, generally from some imperfection in the operation. The accidents, local or general, resulting from the operation, are, according to M. Briquet, very rare. The *local* accidents are chronic inflammation set up in the vessel, to a greater or less extent. The *general* ones are diffuse phlebitis, which may spread to the vessels of the trunk, or even to the heart itself, ending fatally, under the masked form of ataxic fever. "This accident, however, is fortunately very rare. I have only seen it twice happen in upwards of sixty cases operated on." This result is very different from that which has hitherto taken place in England. Hodgson reports the operation as not seldom fatal.

*M. Beclard's Mode of Operating.* The lower part of the thigh or the upper part of the leg is chosen, where the vein is superficial and single. A longitudinal fold of skin is then pinched up, and an incision made into it, which exposes the vessel. A probe, with a double ligature, is then passed under the vein, which is tied and cut above the thread. The parts are then immediately brought together and kept so, in order to secure union by the first intention, if possible. The strictest rest is necessary after the operation. It is well known that Mr. Brodie performed the operation by cutting the vein under the skin, and thus preventing the exposure of the vessel to the air, which he supposed was prejudicial to the success of the process. M. Beclard repeated this operation, but found that phlebitis succeeded as often as after the other mode of operating above described. When there is a packet, as it were, of varicose veins, a longitudinal or a transverse section of the varix, without any ligature, has been successfully practised by Petit, Richerand, and Beclard.

14. *Extirpation.* This consists of two incisions, within which the varicose tumour is included, and then the varix itself is dissected out. It was often practised by the ancients—is very painful—and little, if at all, employed by modern surgeons.

In respect to the consequences of the operations for phlebectasia, as phlebitis, erysipelas, &c. they must be combated by leeches and other antiphlogistic means, which are now well understood.

